# Impact of Community-Based Health Education and Sanitation Interventions on *Opisthorchis viverrini* Infection in an Endemic Area of Northeastern Thailand

**DOI:** 10.3390/ijerph23050553

**Published:** 2026-04-24

**Authors:** Parichart Boueroy, Nattamol Phetburom, Birabongse Hardthakwong, Ratanee Kammoolkon, Panchamapohn Rattanahon, Peechanika Chopjitt, Narita Fakkaew, Pathanan Suwannaboon, Chavanakorn Krueakaew, Patiwat Yasaka, Janjira Hantakhu, Kulthida Y. Kopolrat

**Affiliations:** 1 Faculty of Public Health, Kasetsart University, Chalermphrakiat Sakon Nakhon Province Campus, Sakon Nakhon 47000, Thailand; parichart.bou@ku.th (P.B.); nattamol.phet@ku.th (N.P.); birabongse.h@ku.th (B.H.); rattanee.k@ku.th (R.K.); panchamapohn.r@ku.th (P.R.); peechanika.c@ku.th (P.C.); narita.fak@ku.th (N.F.); pathanan.s@ku.th (P.S.); chavanakorn.kr@ku.th (C.K.); 2 Faculty of Management Technology, Rajamangala University of Technology Isan, Surin Campus, Surin 32000, Thailand; patiwat.ya@rmuti.ac.th; 3 Ban Huai Lua Subdistrict Health Promoting Hospital, Huai Lua Subdistrict, Ban Muang District, Sakon Nakhon 47140, Thailand; kea_janjira0216@hotmail.com

**Keywords:** *Opisthorchis viverrini*, helminth, sand-drying bed system, community-based health education

## Abstract

**Highlights:**

**Public health relevance—How does this work relate to a public health issue?**

- *Opisthorchis viverrini* remains a public health challenge in Southeast Asia, particularly in rural communities of Northeast Thailand, where traditional consumption of raw or undercooked fish sustains transmission.
- Environmental sanitation and inadequate management of fecal sludge may contribute to the persistence and spread of helminth infections in endemic communities.

**Public health significance—Why is this work of significance to public health?**

- Liver fluke infection is associated with serious health outcomes, including hepatobiliary disease and increased risk of cholangiocarcinoma, making prevention and control a critical public health priority in endemic regions.
- Community-based health education combined with environmental sanitation measures can reduce infection prevalence and improve knowledge related to parasite prevention.

**Public health implications—What are the key implications or messages for practitioners, policy makers and/or researchers in public health?**

- Integrated interventions addressing behavioral risk factors (e.g., raw fish consumption) and environmental sanitation should be strengthened in endemic rural areas.
- Public health practitioners and policymakers should promote sustainable community education programs and improved fecal sludge management systems to interrupt the transmission cycle of liver fluke infection.

**Abstract:**

*Opisthorchis viverrini* infection remains a significant public health concern in Southeast Asia, particularly in rural communities of Northeast Thailand, where persistent environmental and behavioral factors sustain transmission. A quasi-experimental study aimed to identify environmental and behavioral risk factors for infection and to evaluate the effectiveness of a community-based intervention program. The intervention program study was conducted over 10 months and comprised three phases: baseline survey‚ health education intervention program implementation‚ and follow-up evaluation. The results were analyzed for the prevalence of parasitic infections, and multivariable logistic regression was performed to identify associated factors. The majority of study participants were female (67.94%)‚ aged 55 to 64 years (48.09%)‚ and farmers (89.31%). Parasitic infections‚ especially *O. viverrini*‚ substantially decreased during the follow-up period‚ and independent risk factors predicting infection included lower education‚ previous infection‚ raw fish consumption‚ and pesticide use‚ according to multivariable logistic regression analysis. This intervention considerably improved knowledge; mean knowledge score increased by 6.29 points (*p* < 0.001). Analysis of fecal sludge after treatment with the sand-drying system identified *S. stercoralis* larvae (20 eggs/L) and *Taenia* spp. eggs (12.4 eggs/g). These findings indicated that, despite treatment, integrated behavioral and environmental interventions can be effective in interrupting parasite transmission in rural endemic settings.

## 1. Introduction

Foodborne trematodes, along with soil-transmitted helminthiases, are the most common neglected tropical diseases (NTDs) in Southeast Asia, predominantly affecting marginalized populations [1]. These infections are closely linked to poverty, inadequate sanitation, and entrenched cultural practices, and they frequently go undiagnosed or underreported. A major NTD endemic in Southeast Asia is infection with the liver fluke *Opisthorchis viverrini* [2,3]. In highly endemic communities, the prevalence of *O. viverrini* infection can reach up to 70%, and approximately 10 million people are estimated to be infected across the region [4,5]. Long-term infection with the parasite can lead to chronic inflammation, hepatobiliary morbidity, and eventually cholangiocarcinoma (CCA) [6,7]. Since 1994, infection with *O. viverrini* has been designated as a group one carcinogen [8], causing bile duct cancer or CCA in humans. Liver fluke-associated CCA is a leading cause of cancer-related mortality in parts of northeast Thailand and neighboring countries, with incidence rates among the highest reported globally. The disease is typically diagnosed at an advanced stage due to nonspecific early symptoms and limited access to effective screening and diagnostic tools, resulting in high case-fatality rates [9]. Therefore, a comprehensive strategy for the prevention, control, and elimination of *O. viverrini* is a prerequisite step toward reducing the incidence of CCA [10].

Despite control efforts, the prevalence of infection persisted after chemotherapy, presumably due to unchanging behavioral and environmental factors [11,12]. *O. viverrini* is a zoonotic parasite whose transmission is sustained through complex, tightly linked interactions between humans, animals, and freshwater ecosystems [2]. A prospective risk factor for *O. viverrini* infection is the consumption of raw or undercooked freshwater fish containing infective metacercariae [13,14,15]. Traditional dishes prepared from raw or partially fermented fish, such as koi pla, pla som, and related preparations, are deeply embedded in the food culture of northeast Thailand and the broader lower Mekong region [16,17]. These dishes are often associated with social gatherings, identity, and cultural heritage, which makes behavior change particularly challenging.

**Figure 1:**
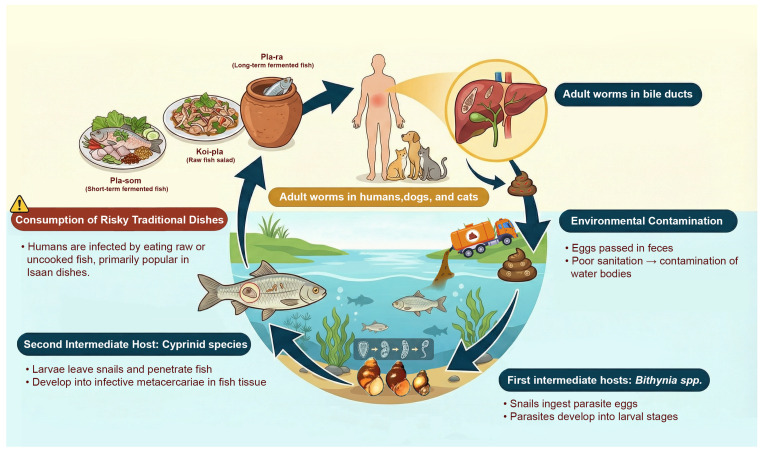
Life cycle of *Opisthorchis viverrini*.

Environmental conditions, including human-induced modifications, play a crucial role in the life cycle of *O. viverrini*, which requires three successive hosts and several morphological transformations. In areas with inadequate sewerage infrastructure and poor sanitation, *O. viverrini* eggs are mainly shed in human feces into freshwater environments, where the first intermediate host, snails of the genus *Bithynia* then ingest them. Infected snails can shed hundreds of cercariae into the water per day [18]. When cercariae encounter the second intermediate host, cyprinid fish, they penetrate the scale of the fish and encyst, forming metacercariae. Moreover, the expansion of aquaculture, irrigation schemes, and reservoir construction may modify freshwater habitats in ways that favor snail and fish populations, thereby increasing opportunities for transmission (Figure 1) [4,19].

Despite prospective control efforts, the prevalence persisted due to reinfection following chemotherapeutic control [20,21]. Widespread drug treatment may also lead to complacency, resulting in continued consumption of raw fish to perpetuate *O. viverrini* transmission [22]. As demonstrated by integrated control initiatives such as the Lawa model, combining health education with environmental management can substantially reduce transmission [23]. Nevertheless, important gaps remain regarding how such approaches can be effectively adapted to different sociocultural and ecological contexts, particularly at the community level, where risk behaviors are deeply rooted.

Therefore, the primary prevention strategy for CCA in liver fluke-endemic areas is the identification and treatment of individuals infected with *O. viverrini* [9,10,11]. Moreover, this study builds on existing integrated control frameworks by providing new empirical insights into the effectiveness of a context-specific, community-based intervention that explicitly links (i) context-specific health education and risk communication that address cultural norms and risk perceptions; and (ii) sanitation and environmental management to reduce contamination of water bodies.

An integrated One Health approach linking human behavior, animal hosts, and environmental hygiene has proven effective in preventing and controlling opisthorchiasis [24]. Therefore, urgent integration of *O. viverrini* control is needed to address both behavioral and environmental factors, thereby interrupting parasite transmission at every life-cycle stage. Thus, the objective of the present study was to identify the risk factors associated with *O. viverrini* infection and to evaluate the effectiveness of an intervention program in rural communities in Thailand.

## 2. Materials and Methods

### 2.1. Study Design and Sample Population

A quasi-experimental study was conducted from February 2024 to January 2025 in an endemic area for *O. viverrini* and helminth infections in Ban Muang District, Sakon Nakhon Province, Northeastern Thailand. Participants were recruited from an endemic community using a community-based approach. The inclusion criteria were: (1) Male and female residents aged 18 years or older, (2) residing in the study area during the project operation, and (3) being in good general health. After obtaining written informed consent, participants were interviewed using a structured questionnaire to collect demographic and lifestyle information, including socio-demographic characteristics, history of *O. viverrini* infection, prior praziquantel treatment, and other health-related factors. Smoking status was classified as never smoker (individuals who had never smoked), former smoker (those who had quit at least 12 months prior to the survey), and current smoker (those who reported smoking within the past 30 days). Alcohol consumption was categorized as never drinker (individuals who had never consumed alcohol), former drinker (no alcohol use within the past 12 months), and current drinker (any alcohol consumption within the past 30 days).

To minimize interviewer bias, all research assistants underwent standardized training and followed a uniform interview protocol with pre-specified question wording and neutral probing techniques. To reduce social desirability bias, interviews were conducted individually in a private setting, participants were assured of confidentiality and anonymity, and they were informed that responses would not affect access to services or benefits. Written informed consent was obtained prior to participation. All complete questionnaires were reviewed for completeness and accuracy before data entry and analysis to ensure data quality and consistency.

### 2.2. Sample Size Calculation

The sample size calculation was estimated using a single-proportion formula:

$$n=\frac{Z\propto /2^{2}\;[p\left(1-p\right)]}{e^{2}}$$

where *p* represents the estimated prevalence of *O. viverrini*, based on prior data indicating a prevalence of 12.2% [25], *Z* is the level of confidence (1.96), and the precision of the estimate (*e*) was set at 5%, yielding a minimum sample size of 112 individuals. To account for possible non-response or participant dropout, an estimated attrition rate of 15% was applied. After this modification, 130 participants were ultimately determined to be the required sample size to maintain statistical power.

### 2.3. Intervention Activities

The 10-month duration was selected to allow sufficient time for repeated health education activities, the adoption and reinforcement of behavioral changes, and the assessment of changes in infection status, including potential reinfection after treatment [26,27]. The experimental study consisted of three stages over these 10 months. Stage 1 involved a baseline survey to assess parasitic infections among 131 participants and to identify associated risk factors. Participants diagnosed with *O. viverrini* infection at baseline were treated with praziquantel according to standard clinical guidelines. In addition, environmental assessments were conducted to evaluate sewage and wastewater management practices in the sand-drying bed system. Stage 2 involved implementing a communication strategy to prevent opisthorchiasis by addressing both clinical and environmental factors in high-risk areas. Stage 3 involved evaluating the experiment using an instrument adapted from the health behavior evaluation form for opisthorchiasis developed by the Department of Disease Control, Ministry of Public Health, Thailand. Meetings with participants were held to inform them about the biology of intestinal parasites, their means of transmission, and strategies to prevent intestinal parasitosis. Recruitment involved obtaining written informed consent to collect stool samples from each participant before and after the intervention.

### 2.4. Formalin-Ethyl Acetate Concentration Method

Fecal samples (each approximately 5 g) were collected from each participant and transferred to the laboratory within 1 day of collection. In the laboratory, each fecal sample was processed for parasite examination using the formalin-ethyl acetate concentration technique (FECT). The FECT procedure was performed as previously described [28]. Briefly, 2 g of fresh stool were fixed in 10% formalin, thoroughly shaken, and strained through gauze. Then 3 mL of ethyl acetate was added to the mixture to extract fat from the feces. After vigorous shaking and centrifugation at 2500 rpm (769× *g*) for 5 min, the supernatant was discarded, and the remaining matter was resuspended in 10% formalin. A drop of the fecal suspension was placed on a slide and examined.

### 2.5. Fecal Sludge and Wastewater Sample Collection and Processing

Fecal sludge and wastewater samples were collected from a sand-drying bed system in Huai Lua Subdistrict. Helminth egg contamination in the sludge samples was examined using simple centrifugal sedimentation. In brief, 20 g of each sample was mixed with 175 mL of distilled water and 75 mL of 5% sodium hypochlorite solution and then observed under a microscope. Helminth egg contamination in wastewater samples was examined using simple centrifugal sedimentation. In brief, 1 L of the water sample was centrifuged in a sedimentation tube for at least 12 h, then rinsed with 0.1% Triton X-100 (Kemaus Elago Enterprises Pty Ltd., Sydney, Australia). Next, a 200 mL sample was transferred to a centrifuge tube and centrifuged at 1000× *g* for 15 min. In addition, any helminth egg contamination in the fecal sludge and wastewater samples was confirmed using the FEST and flotation methods. Representative helminth eggs were observed under a light microscope (Olympus CX43, Tokyo, Japan).

### 2.6. Statistical Analysis

Demographic information and responses to questions about potential risk factors from the questionnaires and laboratory data were entered into an Excel worksheet (Microsoft) and analyzed using SPSS 26 (IBM, Chicago, IL, USA). Baseline characteristics of the sample were presented as frequency numbers and percentages for categorical data. Univariable logistic regression was run to determine the association between *O. viverrini* infection and questionnaire responses, including gender, age, educational levels, occupational status, history of *O. viverrini* infection, history of praziquantel treatment, family history of liver cancer, smoking history, alcohol consumption history, history of raw fish eating, and pesticide usage. Subsequently, factors for which the univariable logistic regression models had a *p*-value < 0.25 were included in a multivariable logistic regression model. A backward variable selection method was used to determine the most parsimonious model. Logistic regression analysis was conducted under the assumptions of a binary outcome variable, independence of observations, and a linear relationship between continuous predictors and the logit of the outcome. Model fit was assessed using the Hosmer–Lemeshow goodness-of-fit test and overall model significance. Multicollinearity among independent variables was assessed using variance inflation factors (VIF) and tolerance statistics. All predictors demonstrated VIF values ranging from 1.05 to 1.64 and tolerance values between 0.61 and 0.95, indicating no evidence of multicollinearity. The adjusted odds ratio (aOR) with a 95% confidence interval (CI) was used to assess the strength of association between variables. Statistical significance was considered at the 95% confidence level (*p* < 0.05). McNemar’s chi-squared test was used to compare the prevalence of *O. viverrini* and other parasitic infections between baseline and follow-up assessments. Moreover, boxplots were constructed to compare the distribution of pre- and post-test knowledge scores visually, and McNemar’s chi-squared test was applied.

## 3. Results

### 3.1. Demographic Characteristics of Participants

In total, 131 participants were sampled, with 67.9% female representation. The majority were aged 55–64 years (48.1%), followed by those aged 45–54 years (35.1%). In addition, over one-half of the participants had completed primary school (51.9%). Agriculture was the predominant occupation (89.3%), with only 10.7% working in other job sectors. Of the respondents, 30.5% reported alcohol consumption, while 12.2% reported smoking, whereas the majority (87.8%) had no smoking history. Regarding raw fish consumption, most participants reported a history of consuming raw fish, with 79.4% having eaten raw fish (Table 1).

**Figure 2:**
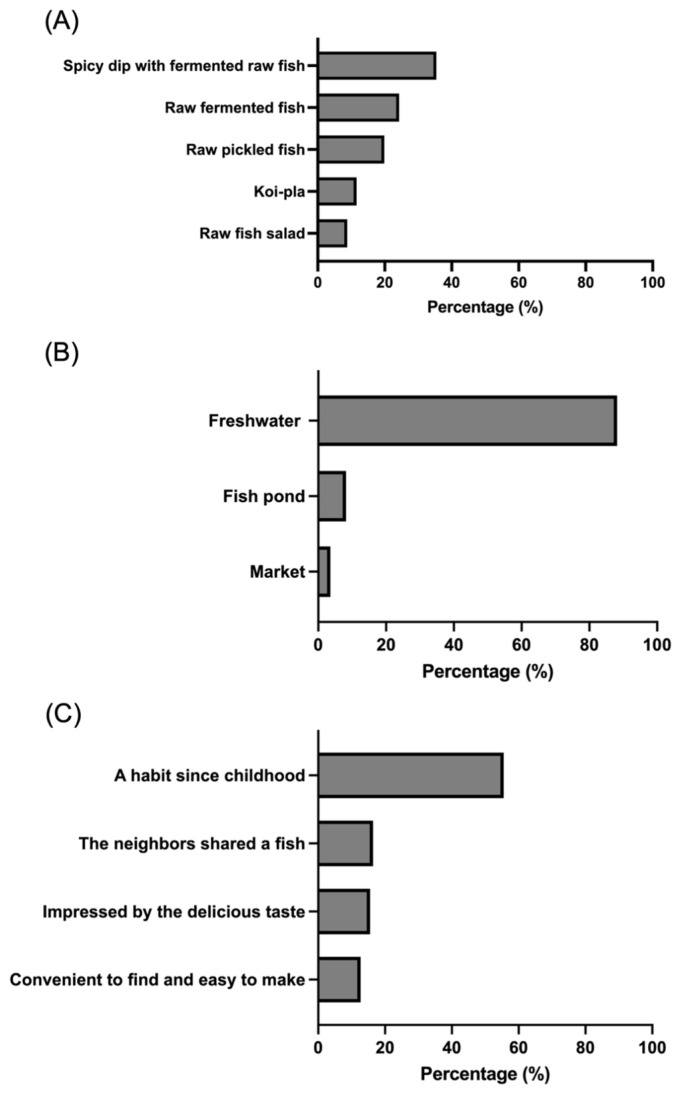
Data from project participants regarding varieties of raw fish (**A**), sources of cyprinid fish (**B**), and motivations for consuming raw fish (**C**).

In terms of raw fish consumption patterns and sources, the predominant dish among raw fish consumers was spicy dip with fermented raw fish (35.4%), followed by fermented raw fish (24.3%) and pickled raw fish (19.9%), respectively (Figure 2A). The majority of the raw fish consumers obtained their fish from natural freshwater sources (88.2%), while a smaller proportion sourced fish from fishponds (8.2%) or local markets (3.6%) (Figure 2B). Habitual practice was the primary motivation for raw fish consumption, with 55.5% reporting that eating raw fish had been a tradition since childhood. Additional reasons included sharing fish with neighbors (16.4%), preference for taste (15.5%), and availability and convenience (12.7%), respectively (Figure 2C).

### 3.2. Baseline of Intestinal Parasite Prevalence Determined Based on Fecal Examination

**Figure 3:**
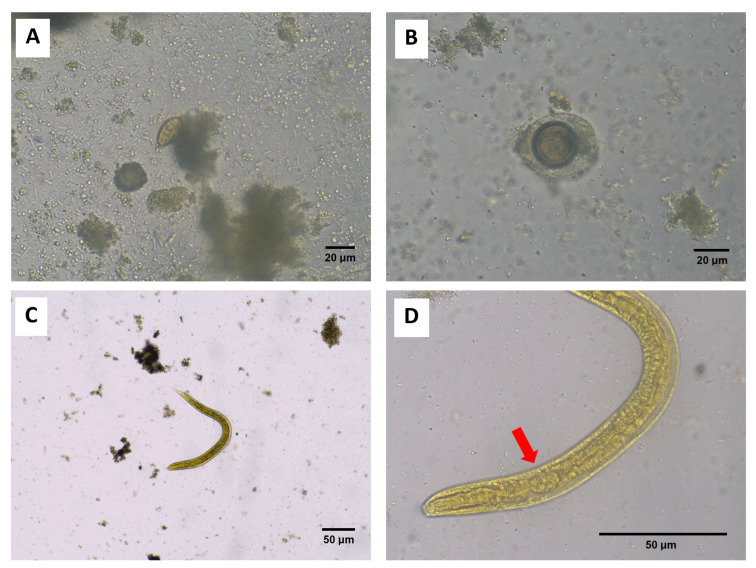
Light microscopic images of isolated parasites were identified in stool samples. (**A**) *O. viverrini* (40× magnification), (**B**) *Taenia* spp. (40× magnification), (**C**) A rhabditiform larva of *S. stercoralis* (10× magnification), and (**D**) rhabditiform larva of *S. stercoralis* (40× magnification), showing characteristic rhabditiform esophagus (red arrow).

Based on the FECT examination, the overall prevalence of intestinal parasites was 21.4% (28 of 131). The most common species was *O. viverrini*, with an overall prevalence of 19.1%. This was followed by infections with *Strongyloides stercoralis*, with a prevalence of 0.8%, as well as *Taenia* spp., and double infection between *Taenia* spp. and *S. stercoralis*, with a prevalence of 0.8% each (Figure 3A–D).

### 3.3. Risk Factors Associated with O. viverrini Infection

Table 2 presents the univariable and multivariable logistic regression analyses conducted to identify factors associated with *O. viverrini* infection among participants. After adjusting for potential confounders, three factors remained significantly associated with *O. viverrini* infection (*p* < 0.05). Participants with secondary education or higher had substantially greater odds of infection than those with only primary education (aOR = 2.68, 95% CI: 1.08–6.65, *p* = 0.033). A history of previous *O. viverrini* infection was also significantly associated with current infection (aOR = 3.12, 95% CI: 1.09–8.98, *p* = 0.034). In addition, individuals who reported consuming raw fish had an increased risk of infection (aOR = 4.59, 95% CI: 1.00–20.99, *p* = 0.049), while pesticide usage showed a strong association with infection (aOR = 3.77, 95% CI: 1.28–11.17, *p* = 0.016). Other variables (gender, age group, occupation, history of praziquantel treatment, family history of liver cancer, smoking, alcohol consumption) were not significantly associated with *O. viverrini* infection.

### 3.4. Effectiveness of Community Intervention

**Figure 4:**
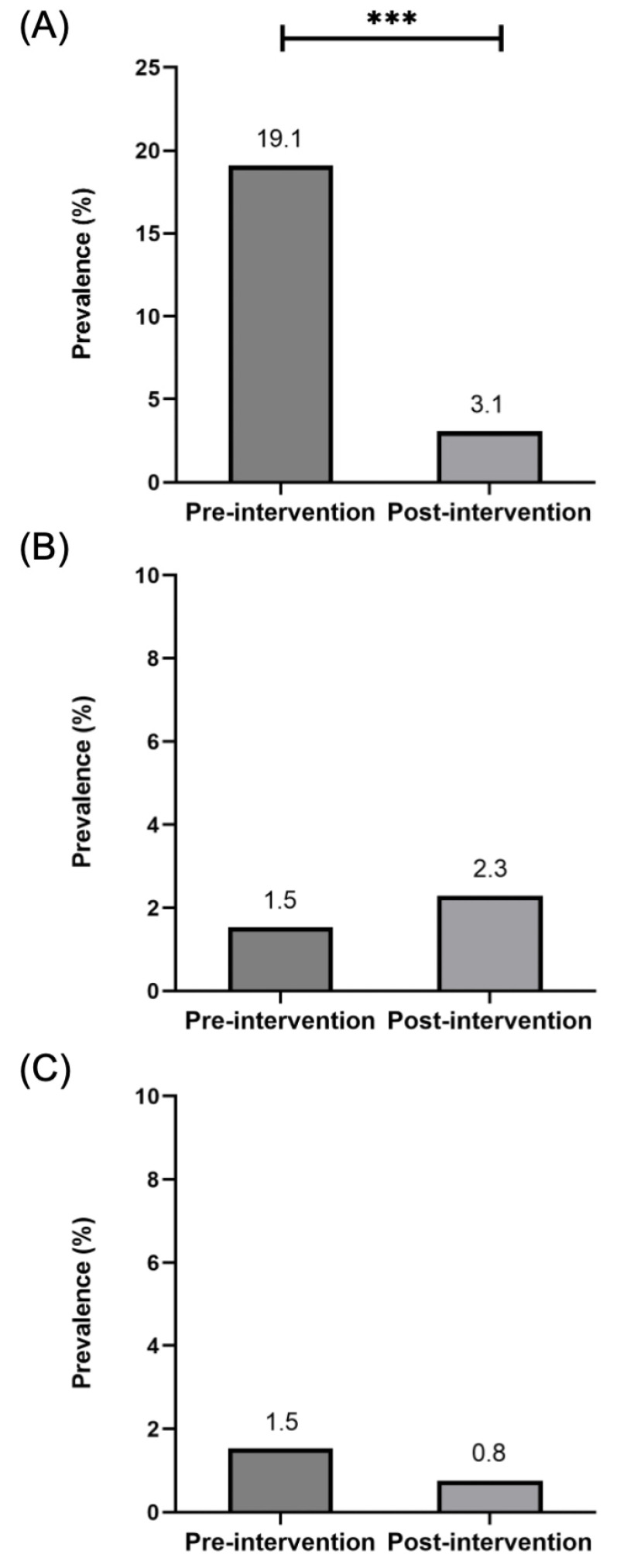
Prevalence of *O. viverrini* (**A**), *S. stercoralis* (**B**), and *Taenia* spp. (**C**) infections among participants before and after intervention (*n* = 131), based on McNemar’s chi-squared test. *** *p* < 0.001.

The overall prevalence of infection significantly declined after the intervention, from 21.4% (28/131) at baseline to 6.1% (8/131) post-intervention. Following the health education program, the prevalence of *O. viverrini* significantly decreased (Figure 4A). In contrast, the prevalence of *S. stercoralis* increased slightly, from 1.5% at baseline to 2.3% at follow-up (Figure 4B). Meanwhile, the prevalence of *Taenia* spp. decreased from 1.5% at baseline to 0.8% after the intervention (Figure 4C). No parasite eggs were detected in the wastewater or fecal sludge samples collected post-intervention (Table 3).

### 3.5. Knowledge Levels Before and After Intervention

**Figure 5:**
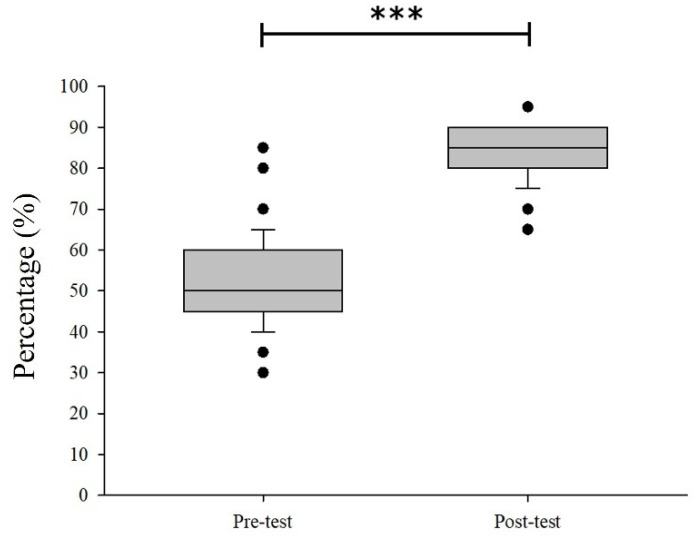
Boxplot comparing pre- and post-test knowledge scores regarding *O. viverrini* infection among participants. Scores increased markedly following the intervention, indicating improved knowledge levels, as determined by McNemar’s chi-squared test. *** *p* < 0.001.

Figure 5 compares knowledge levels regarding *O. viverrini* infection before and after intervention among the 131 participants. At baseline, participants demonstrated limited knowledge, with most classified in the low (52.67%) or medium (46.56%) categories, and only 0.76% achieving a high level of knowledge. The mean pre-test score was 10.43 (SD = 1.96), ranging from 6 to 17. Following the intervention, there was a substantial improvement in knowledge. All participants moved out of the low-knowledge category, with 58.78% achieving high scores and 41.22% categorized as having medium knowledge. The average post-test score increased to 16.72 (SD = 1.35), with a range from 13 to 19 (Table S1).

## 4. Discussion

This study demonstrated the efficacy of an integrated approach in reducing the burden of *O. viverrini* and intestinal parasitic infections, while concurrently improving health-related knowledge among individuals residing in an endemic area. The study comprised predominantly middle-aged and older agricultural workers with low levels of formal education. These demographic characteristics were consistent with other published reports, indicating that *O. viverrini* infection remained concentrated among older adults in agrarian regions of Northeast Thailand, where traditional food practices persist [14,25]. Importantly, focusing on older individuals may enhance the public health relevance of the findings, as this group represents a key reservoir of infection in endemic settings and is critical for interrupting transmission at the community level. In addition, the community-based nature of this study may have facilitated greater participation among older adults, who are generally more available and more engaged in local health activities. A key finding was the high prevalence of at-risk behavior, with more than half of the participants reporting consumption of raw or fermented fish dishes, particularly spicy fermented fish dip, a practice deeply rooted in cultural and community traditions. Similar research has highlighted the consistently strong influence of social norms and familial practices on raw-fish consumption in endemic regions [16].

The initial prevalence of intestinal parasites was 21.4%, with *O. viverrini* accounting for 19.1%. This aligned with a report showing ongoing endemicity in specific rural communities despite decades of national control efforts [25]. Although infections with *S. stercoralis* and *Taenia* spp. are less common, *O. viverrini* remains the biggest parasitic health threat in the region, especially given its established link to cholangiocarcinoma [9,25]. Among risk factors, eating raw fish significantly increased the odds of infection, consistent with existing epidemiological evidence [11,14]. Notably, participants with higher levels of education reported a higher risk of infection than those with only primary education. This contrasted with expected trends but supported another report that education alone is insufficient to change culturally reinforced dietary behaviors in endemic communities [16]. The history of prior infection predicted current infection, indicating ongoing exposure and persistent behavioral patterns, consistent with another reinfection study [20]. The link between pesticide use and *O. viverrini* infection, although statistically significant, may reflect underlying occupational and environmental exposures rather than a direct causal relationship. Farmers who apply pesticides often work in or near rice paddies and wetlands where the parasite is endemic, thereby increasing their risk of exposure. In addition, the consumption of raw or undercooked fish (e.g., koi-pla) during field activities is a well-established behavioral risk factor that may further contribute to the observed patterns of infection [29].

Praziquantel, administered at baseline to infected individuals, is highly effective, with reported cure rates ranging from approximately 88% to 100% within 4 weeks of treatment [21]. However, the substantial reduction in infection prevalence observed in this study from 21.4% to 6.1% is more appropriately interpreted as the combined effect of pharmacological treatment and the integrated intervention, including health education and environmental sanitation [30]. The absence of parasite eggs in wastewater and sludge following the intervention indicated a lower risk of community-level transmission. Furthermore, the notable improvement in knowledge scores underscores the importance of targeted health education to raise awareness and promote preventive behaviors, consistent with other community-based programs in Thailand [23,30,31]. The helminth eggs in wastewater and fecal sludge samples from the sand-drying bed system were examined. Among the helminth eggs identified in the fecal sludge were *S. stercoralis* larvae (20 eggs/g) and *Taenia* spp. eggs (12.4 eggs/g). These findings indicated that, despite treatment, viable helminth eggs can persist in fecal sludge if the system is not properly maintained, posing a potential health risk [32,33]. There were significant differences in helminth egg contamination in the fecal sludge samples after interventions to reduce system overload and to maintain the sand-drying bed system to improve sedimentation and dehydration, which are the main mechanisms in planted drying beds that result in pathogen reduction in fecal sludge [34]. However, the helminth eggs were not identified in wastewater samples. Notably, no helminth eggs were detected in the wastewater samples, suggesting that the drying bed system effectively reduced parasite contamination in the liquid effluent, consistent with another study demonstrating the efficacy of drying beds in pathogen reduction [35]. However, the presence of helminth eggs in fecal sludge indicates incomplete pathogen removal in the solid fraction that may result from inadequate drying, overloading, or poor maintenance. The link between intestinal parasite contamination and insufficient maintenance of the drying bed system underscores the critical role of regular system upkeep in ensuring optimal pathogen reduction [36]. Successful intervention, as shown here, depends not only on installation but also on the system administrators’ ongoing understanding and commitment to maintaining the drying beds annually, thereby reducing health risks associated with fecal sludge reuse or disposal [37].

Prior to the intervention, the majority of participants demonstrated low (52.67%) to moderate (46.56%) levels of knowledge, with only 0.76% attaining a high classification. These findings reflected a generally limited baseline understanding of the health topic under assessment. Following the intervention, there was a substantial shift in knowledge levels, with none of the participants remaining in the low knowledge category. Notably, over one-half of the participants (58.78%) achieved high knowledge scores post-intervention, while 41.22% fell within the medium range. The mean post-test score increased to 16.72 (SD = 1.35), with a narrower score range of 13 to 19, indicating both overall improvement and reduced variability in knowledge outcomes. This marked improvement in knowledge suggested that the intervention effectively enhanced participants’ understanding of the targeted health concepts. The increase in both the mean score and the proportion of participants achieving high levels of knowledge aligned with other studies that highlighted the effectiveness of structured health education programs [30]. Such knowledge gains are critical, as improved health literacy has been linked to better health behaviors and outcomes, including the prevention of infectious diseases such as *O. viverrini* infection [38]. These findings are consistent with other published research, indicating that structured health education increases knowledge and promotes health literacy [39]. Similarly, Patchasuwan et al. [40] found that participatory action research using school-based interventions improved health literacy and preventive practices against liver fluke in endemic regions. Sustaining these knowledge improvements through ongoing education and supportive environments will be key to achieving long-term health benefits [39].

Despite these promising results, several limitations should be acknowledged. The study was conducted in a single community and relied on self-reported behaviors, which may be subject to social desirability bias, particularly following the intervention. In addition, the absence of individual-level parasitological follow-up to confirm cure after treatment limits the ability to distinguish between treatment failure and reinfection. Furthermore, the quasi-experimental design without a control group limits causal inference, as unmeasured confounding and external influences cannot be fully excluded. In addition, longitudinal follow-up is required to assess the sustainability of behavioral changes and to track reinfection rates over time. Thus, it will be essential for ongoing culturally tailored health promotion, regular stool surveillance, and policy-supported environmental sanitation efforts to sustain progress toward *O. viverrini* elimination.

## 5. Conclusions

This study demonstrated the effectiveness of an integrated intervention in reducing *O. viverrini* infection and improving health-related knowledge among participants in an endemic area. The prevalence of *O. viverrini* infection significantly declined following the intervention, highlighting the success of targeted health education and environmental sanitation strategies. In addition, the intervention led to a substantial, statistically significant increase in participants’ health-related knowledge. This finding supports the role of community-based educational programs in improving health knowledge, a critical factor in promoting behavioral change and reducing infection risk. A multidisciplinary approach integrating public health, education, and environmental management is essential to control liver fluke transmission and to protect communities from long-term health consequences such as cholangiocarcinoma. Furthermore, the results highlight the potential for policy-level adoption and the sustainability of community-led initiatives, ensuring continued impact beyond the study period.

## Figures and Tables

**Table 1:** Baseline characteristics of project participants (*n* = 131).

Characteristic	Number (*n*)	Percentage (%)
Gender		
Male	42	32.1
Female	89	67.9
Age (years)		
<45	9	6.9
45–54	46	35.1
55–64	63	48.1
≥65	13	9.9
Education		
Primary School	68	51.9
Junior high school	26	19.8
Senior high school and higher	37	28.2
Occupation		
Agriculture	117	89.3
Other	14	10.7
Alcohol consumption		
Yes (current or former)	40	30.5
Never	91	69.5
Smoking history		
Yes (current or former)	16	12.2
Never	115	87.8
History of raw fish eating		
Yes (current or former)	104	79.4
Never	27	20.6

**Table 2:** Demographic and lifestyle factors associated with *O. viverrini* infection were assessed using FECT in Ban Muang District, Sakon Nakhon Province, Thailand, with univariable and multivariable logistic regression (*n* = 131).

Factor	Number Examined	*O. viverrini*		OR	aOR	95% CI	*p*-Value
		Positive *n* (%)	Negative *n* (%)				
Gender							
Female	89	16 (18.0)	73 (82.0)	1.83	1.71	0.702–4.165	0.238
Male	42	12 (28.6)	30 (71.4)	1.00	1.00		
Age groups (years)							
≥55	76	18 (23.7)	58 (76.3)	1.40	1.22	0.496–2.995	0.667
<55	55	10 (18.2)	45 (81.8)	1.00	1.00		
Educational levels							
Secondary	63	18 (28.6)	45 (71.4)	2.32 *	2.68	1.082–6.645	**0.033**
Primary	68	10 (14.7)	58 (85.3)	1.00	1.00		
Occupational status							
Farmer	117	27 (23.1)	90 (76.9)	3.90	3.68	0.457–29.653	0.221
Non-farmer	14	1 (7.1)	13 (92.9)	1.00	1.00		
History of *O. viverrini* infection							
Yes	19	8 (42.1)	11 (57.9)	3.35 **	3.12	1.087–8.978	**0.034**
Never	112	20 (17.9)	92 (82.1)	1.00	1.00		
History of praziquantel treatment							
Once and over	18	5 (27.8)	13 (72.2)	1.51	1.46	0.465–4.599	0.516
Never	113	23 (20.4)	90 (79.6)	1.00	1.00		
House near a water source							
Yes	31	7 (22.6)	24 (77.4)	1.10	1.16	0.428–3.119	0.775
No	100	21 (21.0)	79 (79.0)	1.00	1.00		
Family history with liver cancer							
Yes	16	3 (18.8)	13 (81.3)	0.83	0.92	0.239–3.549	0.906
No	115	25 (21.7)	90 (78.3)	1.00	1.00		
Smoking history							
Yes (current or former)	16	2 (12.5)	14 (87.5)	0.49	0.27	0.51–1.391	0.117
Never	115	26 (22.6)	89 (77.4)	1.00	1.00		
Alcohol consumption history							
Yes (current or former)	40	9 (22.5)	31 (77.5)	1.10	0.91	0.369–2.442	0.913
Never	91	19 (20.9)	72 (79.1)	1.00	1.00		
History of raw fish eating							
Yes (current or former)	104	26 (25.0)	78 (75.0)	4.18 *	4.59	1.004–20.999	**0.049**
Never	27	2 (7.4)	25 (92.6)	1.00	1.00		
Pesticide usage							
Yes	17	8 (47.1)	9 (52.9)	4.18 **	3.77	1.275–11.173	**0.016**
No	114	20 (17.5)	94 (82.5)	1.00	1.00		

Goodness-of-fit test model = 0.65, *p* = 0.940; Data presented were analyzed using a logistic regression model showing OR and aOR with 95% CI and *p*-values. *, ** indicate OR with a significance level of *p* < 0.05, *p* < 0.01 and *p* < 0.001, respectively. Bolded font denotes statistical significance at *p* < 0.05.

**Table 3:** Prevalence of parasites in wastewater and fecal sludge in Ban Muang District, Sakon Nakhon Province, Thailand.

Parasite	Wastewater (Eggs/L)		Fecal Sludge (Eggs/g)	
	Pre-Test	Post-Test	Pre-Test	Post-Test
*Taenia* spp.	0	0	12.4	0
*S. stercoralis*	0	0	20	0
*O. viverrini*	0	0	0	0

## Data Availability

The original contributions presented in the study are included in the article; further inquiries can be directed to the corresponding author.

## Supplementary Materials

The following supporting information can be downloaded at: https://www.mdpi.com/article/10.3390/ijerph23050553/s1, Table S1: Knowledge level pre- and post-intervention (n = 131).
