# Supplementary material for: Impact of Community-Based Health Education and Sanitation Interventions on Opisthorchis viverrini Infection in an Endemic Area of Northeastern Thailand
Source: Int J Environ Res Public Health. 2026 Apr 24;23(5):553. doi: 10.3390/ijerph23050553 (PMC13207269; doi:10.3390/ijerph23050553)
Supplement: Supplementary file 1 [file ijerph-23-00553-s001.zip › ijerph-4235916-supplementary.pdf]

Table S1. Knowledge level pre- and post-intervention (n = 131).

Level of knowledge	Pre-test		Post-test	
	N	%	N	%
Low (score 1-10)	69	52.67	0	0.00
Medium (score 11-16)	61	46.56	54	41.22
High (score 17-20)	1	0.76	77	58.78
Mean ± SD	10.43 (S.D.=1.96)		16.72 (S.D.=1.35)	
	Min =6, Max=17		Min=13, Max =19	
